# Cyto-Safe: A Machine Learning Tool for Early Identification of Cytotoxic Compounds in Drug Discovery

**DOI:** 10.1021/acs.jcim.4c01811

**Published:** 2024-12-11

**Authors:** Francisco L. Feitosa, Victoria F. Cabral, Igor H. Sanches, Sabrina Silva-Mendonca, Joyce V. V. B. Borba, Rodolpho C. Braga, Carolina Horta Andrade

**Affiliations:** † Laboratory for Molecular Modeling and Drug Design (LabMol), Faculdade de Farmácia, Universidade Federal de Goiás, Goiânia, Goiás 74605-220, Brazil; ‡ Center for the Research and Advancement in Fragments and molecular Targets (CRAFT), School of Pharmaceutical Sciences at Ribeirão Preto, University of São Paulo, Ribeirão Preto, São Paulo 05508-220, Brazil; § Center for Excellence in Artificial Intelligence (CEIA), Institute of Informatics, Universidade Federal de Goiás, Goiânia, Goiás 74605-170, Brazil; ∥ InsilicAll Inc., São Paulo, São Paulo 04571-010, Brazil

## Abstract

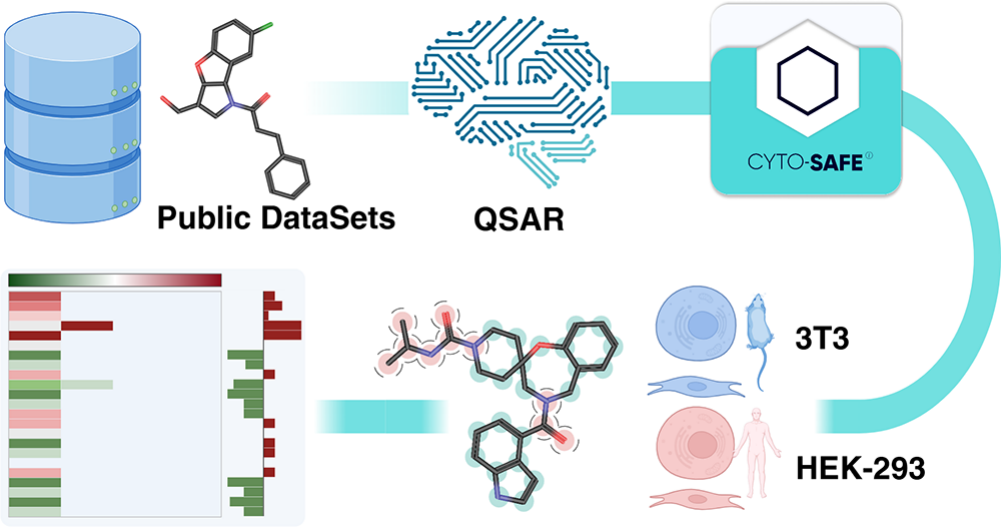

Cytotoxicity is essential in drug discovery, enabling early evaluation of toxic compounds during screenings to minimize toxicological risks. *In vitro* assays support high-throughput screening, allowing for efficient detection of toxic substances while considerably reducing the need for animal testing. Additionally, AI-based Quantitative Structure–Activity Relationship (AI-QSAR) models enhance early stage predictions by assessing the cytotoxic potential of molecular structures, which helps prioritize low-risk compounds for further validation. We present a freely accessible web application designed for identifying potential cytotoxic compounds utilizing QSAR models. This application utilizes machine learning techniques and is built on a data set of approximately 90,000 compounds, evaluated against two cell lines, 3T3 and HEK 293. Users can interact with the app by inputting a SMILES representation, uploading CSV or SDF files, or sketching molecules. The output includes a binary prediction for each cell line, a confidence percentage, and an explainable AI (XAI) analysis. Cyto-Safe web-app version 1.0 is available at http://insightai.labmol.com.br/.

## Introduction

Cell viability and cytotoxicity assays are fundamental tools used in biomedical research to measure the cytotoxic effects of various substances on living cells. Cytotoxicity specifically refers to the detrimental impacts these substances have on cellular health, often leading to cell death.^1^ These assays are critical for drug screening, identifying compounds that demonstrate cytotoxic effects which are then typically excluded in both target-based and cell-based phenotypic screenings. This is particularly vital in the context of oncology and neurodegenerative diseases, where understanding substance toxicity is crucial for drug development.^2^

A significant advantage of *in vitro* assays is their ability to perform high-throughput screening, allowing researchers to efficiently identify toxic compounds or potential therapeutic agents from large numbers of samples. Additionally, these assays align with the growing emphasis on ethical research by reducing the need for animal testing, making them increasingly valuable in the scientific community.^3,4^ They operate by measuring various cellular functions that indicate cytotoxicity, such as cell membrane permeability, enzyme activity, cell adherence, ATP production, coenzyme production, and nucleotide uptake activity.^5^ Accurately predicting chemical-induced cytotoxicity early in the drug development process (preferably before the compounds are even synthesized) is essential. Early detection of cytotoxicity can help prevent costly failures in later stages of development and ensures that only the most promising candidates advance.

The increasing costs associated with ADME/Tox (Absorption, Distribution, Metabolism, Excretion, and Toxicity) studies have spurred the development of *in silico* methods, particularly within large pharmaceutical companies that possess extensive and internally consistent data sets. While initial computational efforts outside the pharmaceutical sector faced limitations due to smaller data sets, the growing availability of larger data sets in public repositories has significantly improved the potential for successful model development.^6^ The use of artificial intelligence (AI) to build cytotoxicity models shows great promise for enhancing early stage cytotoxicity prediction. By predicting the cytotoxic potential of chemical compounds based on their molecular structures, these computational models can support virtual screening campaigns, helping to prioritize compounds with lower cytotoxic risks for further experimental validation. This approach streamlines the identification of viable drug candidates.

Building on insights from our previous research,^7−10^ we have developed QSAR models using a diverse data set of compounds tested on 3T3 and HEK-293 cell lines. We present a new web-accessible application designed to predict the cytotoxicity potential of chemicals, integrating a carefully curated database of 3T3 and HEK-293 cytotoxicity data. The web app features novel models for predicting cytotoxicity, developed exclusively with open-source tools. Available as a web version (version 1.0), it facilitates efficient virtual screening of chemical libraries, aiding in the identification of potential cytotoxic compounds for further investigation. The result includes a binary prediction for each cell line, a confidence percentage, and an explainable AI (XAI) analysis for visual interpretation of the results. This tool can be freely accessed at LabMol Insight AI portal http://insightai.labmol.com.br/.

## CYTO-SAFE

### Data Collection

Cytotoxicity data was sourced from a data set provided by the National Center for Advancing Translational Sciences (NCATS).^11^ This data set includes the results of approximately 90,000 compounds tested for cytotoxicity using the luciferase assay, commercially known as CellTiter-Glo, in a 48 h of incubation time, across two different cell lines: 3T3 and HEK 293. The original data can be accessed via PubChem under AID 1345082 and AID 1345083.

### Data Cleaning and Curation

Initially, both data sets comprised 93,781 records. However, after eliminating entries with inconclusive outcomes and incomplete chemical structure information, the analysis yielded 67,041 compounds from the 3T3 series and 64,508 compounds from the HEK 293 series.

Subsequently, we implemented a rigorous data curation protocol as described by Fouches et al.,^12,13^ resulting in 66,620 unique compounds for the 3T3 series. According to the threshold stablished of EC50 value of ≤10 μM,^11^ 62,613 compounds were labeled as noncytotoxic, and 4,007 records were considered cytotoxic for the 3T3. In the HEK data set, a total of 64,094 records were analyzed, with 6,141 compounds labeled as cytotoxic.

To tackle potential data unbalancing that might introduce bias into the classification models, we strategically applied the NearMiss v.3 under sampling method^14^ executed on Imbalanced-learn package (https://imbalanced-learn.org), setting the sampling strategy as 0.2 and number of near neighbors to 50. This method enabled to get compounds in the majority class (noncytotoxic) with the minimum distance from minority class examples (cytotoxic) maintaining a balanced proportion between cytotoxic and noncytotoxic samples. By adopting this approach, our primary objective was to establish a more equitable and representative data set, thereby enhancing the effectiveness of our classification model training process. The entire processed data is available in Supporting Information.

### QSAR Modeling

Classification QSAR models were generated and validated in accordance with the established standards and principles of QSAR modeling.^15,16^ The molecules were converted into a binary language, based on Extended Connectivity Fingerprints^17^ with radius 2 (ECPF4) and 1024 bits, using the open-source library RDKit.^18^ ECFP was chosen to capture detailed atomic environments without relying on predefined features. Light Gradient Boosting machine learning algorithm (LGBM)^19^ was executed in Python 3.10. The data set was stratified split into training (80%) and external (20%) sets. The external set was held out entirely during hyperparameter optimization to ensure unbiased evaluation. Bayesian optimization was conducted using the scikit-optimize, with 100 iterations and 10-fold cross-validation, optimized by balanced accuracy. Class weights were applied to handle class imbalance in the data set. The selected hyperparameters for the best models are provided in the Supporting Information. For all models, we calculated the following metrics on the external set: Balanced Accuracy (BACC), Matthew’s correlation coefficient (MCC), Precision, Recall, F1 score, and plotted the confusion matrix.

### Y-Randomization

We performed 50 rounds of Y-randomization to assess the robustness and validity of our predictive models. Y-randomization involves shuffling the dependent variable (*Y*; cytotoxicity outcomes) while keeping the independent variables (*X*; molecular fingerprints) intact.

### Deployment

The Cyto-Safe web application was deployed on a cloud-based platform, utilizing a Flask backend and a Jinja2 template-driven frontend to ensure scalability and responsive user interfaces. Machine learning models were then integrated within the Flask framework to facilitate both individual and batch predictions of up to ten compounds via CSV or SDF file inputs. The application incorporates the Ketcher molecular editor (version 2.10.0; EPAM), an open-source web-based chemical structure editor, to provide an intuitive interface for drawing and editing chemical structures, enhancing user experience and data accuracy. Prediction results can be exported as spreadsheets for detailed analysis or as web-based reports for immediate review.

### Explainable AI (XAI)

In this study, we employed an Explainable AI (XAI) framework to interpret our model’s binary classification predictions regarding the cytotoxicity of compounds in 3T3 and HEK-293 cell lines. We used the methodology proposed by Riniker and Landrum^20^ that systematically removes bits in the molecular fingerprints that correspond to specific atoms or functional groups and assess how these changes influenced the model’s predictions. We normalized these contributions and visualized them using similarity maps and heatmaps analogous to topographical representations.

In these visualizations, structural fragments predicted to increase toxicity were highlighted in red, while those predicted to decrease toxicity were highlighted in green. This approach allowed us to identify key structural features affecting the model’s decisions, providing deeper insights into the patterns and potential biases related to the predicted outcome.

## Results and Discussion

### Modeling

The under-sampling technique was applied to the majority class (noncytotoxic) using two different proportions: 1:1 and 1:5, relative to the minority class, cytotoxic. As a result, the balanced 3T3 data set comprised 8,014 records for the 1:1 ratio and 24,042 for the 1:5 ratio. For the HEK 293 balanced data sets, the corresponding records were 12,282 and 36,846, respectively.

Further analysis was conducted using clustering to identify groups of structurally similar compounds that exhibit contrasting outcomes (cytotoxic versus noncytotoxic), with the goal of simulating potential activity cliffs within the training set. The results revealed that only 11.8% of the clusters from the 3T3 data set and 13.2% from the HEK293 data set contained compounds with differing outcomes. These findings indicate a high level of data reliability while also highlighting the challenges that the algorithm must navigate to learn effectively from the training set (see Supporting Information - Supplementary Methods and Results).

Following training, all models were evaluated on the test set. The models exhibited satisfactory performance across both under-sampling proportion ratios, indicating their proficiency in distinguishing between cytotoxic and noncytotoxic compounds. Notably, the models demonstrated robust generalization capabilities by accurately classifying samples not encountered during the training phase.

When comparing overall metrics, the models trained with the 1:5 ratio showed a slight improvement over those using the 1:1 ratio. This was particularly evident in the Matthews Correlation Coefficient (MCC), where the average values increased from 0.61 to 0.86, underscoring the reliability and informativeness of the predictions. Sensitivity (Se) also improved significantly, with average values rising from 0.65 to 0.83, indicating that the models retained their ability to correctly identify cytotoxic compounds despite the increased under-sampling. These results, detailed in Table 1, support the decision to adopt the 1:5 ratio in Cyto-Safe’s back-end prediction algorithm.

**Table 1:** Performance Metrics of QSAR Model Predictions on the Test Sets for Cytotoxicity Classification in Different Cell Lines and Balancing Proportions, Using the LGBM Algorithm^a^

	BACC	AUC	F1	MCC	Precision	Se	Sp
3T3 Unbalanced	0.81	0.81	0.69	0.68	0.78	0.63	0.99
3T3 1:1	0.80	0.80	0.80	0.59	0.80	0.79	0.81
3T3 1:5	0.92	0.92	0.90	0.88	0.96	0.84	0.99
HEK Unbalanced	0.83	0.83	0.73	0.71	0.81	0.67	0.98
HEK 1:1	0.81	0.81	0.81	0.63	0.81	0.81	0.81
HEK 1:5	0.90	0.90	0.87	0.84	0.92	0.82	0.99

a BACC: Balanced accuracy; AUC: Area under the curve; F1: F1 score; MCC: Matthew’s correlation coefficient; Se: Sensibility; Sp: Specificity.

The t-SNE plots for each balanced approach (see Supporting Information) reflect the statistical improvements observed with a 1:5 ratio compared to both the 1:1 ratio and the unbalanced set. Unlike the 1:1 ratio, which excludes highly similar compounds and risks misleading predictions, the 1:5 ratio better captures the chemical space of the original unbalanced data set.

Moreover, we applied the Y-randomization method to determine if the correlations identified by the model between molecular fragments and cytotoxic effects are genuine or simply artifacts of random associations. As a result of the 50 rounds on each data set, we observed an average ACC of 0.5 for both data sets and MCC of 0.02 and 0.01 for 3T3 and HEK 293, respectively, confirming the robustness of the models.

### Usability

**Figure 1:**
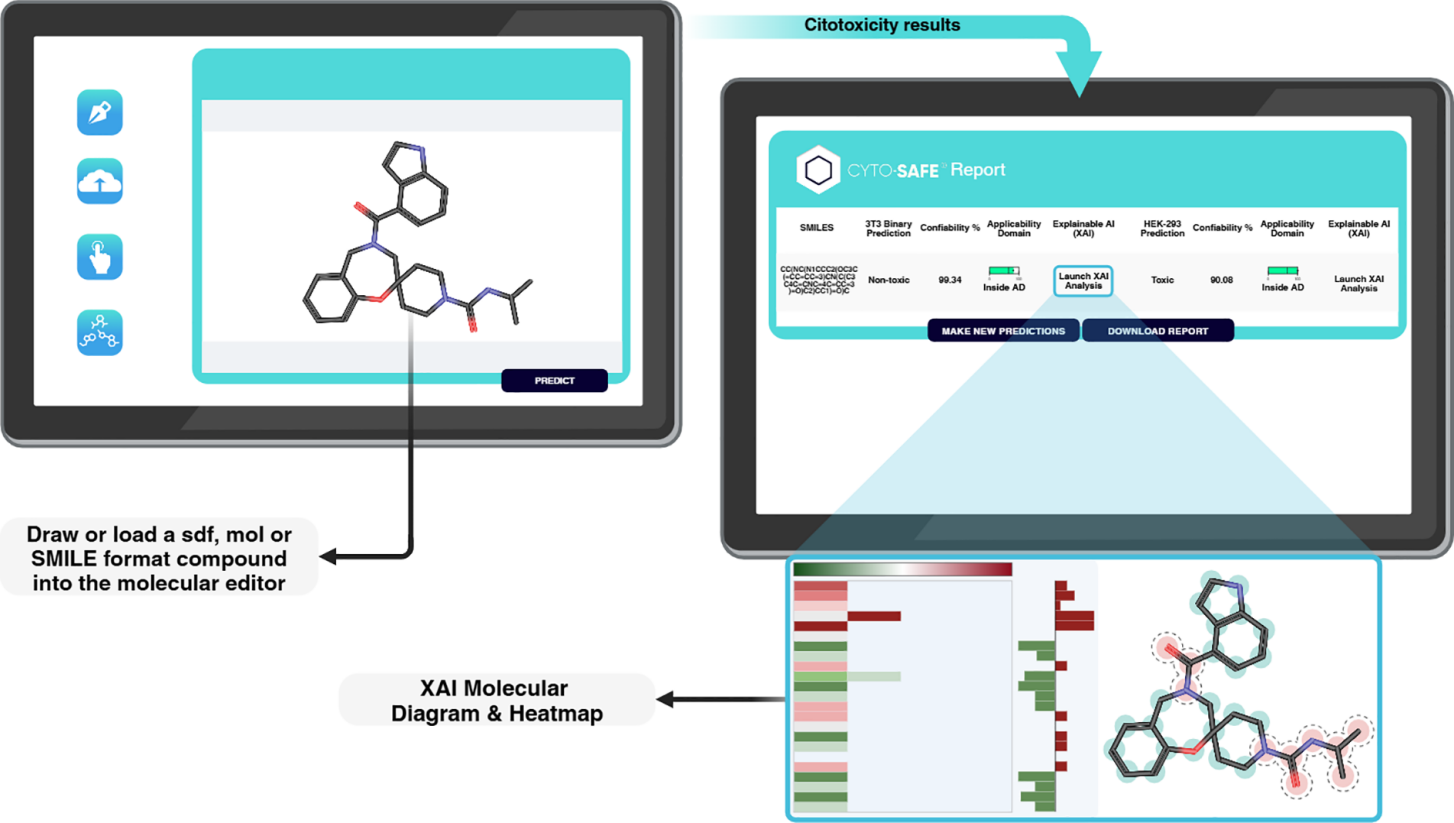
General scheme of usage, outcome and XAI of Cyto-Safe web app.

As mentioned previously, users can easily draw a molecule for testing or upload a batch of molecules for prediction using a CSV or SDF file, with a limit of ten SMILES per request. The results are displayed in a list format for each model (3T3 and HEK 293), indicating whether each molecule is predicted to be toxic or nontoxic. Additionally, the predicted probabilities (representing the model’s confidence) are provided as percentages, calculated using the *predict_proba* method from the LightGBM library. A dedicated button also allows users to initiate Explainable Artificial Intelligence (XAI) analysis (Figure 1).

Furthermore, in accordance with the best practices established by the Organization for Economic Co-operation and Development (OECD),^16^ we have defined the applicability domain of the model’s predictions to ensure their reliability. The threshold was set at 0.09, corresponding to the fifth percentile of the Tanimoto similarity distribution among compounds in the training set.^21^ This relatively low threshold reflects the high structural diversity within the training set. A similarity distribution analysis is provided in Figure S3 (Supporting Information). The information regarding whether the tested compound is within or outside the applicability domain is available on the prediction results page, helping users understand the limitations of the model’s outputs.

### Explainable AI (XAI) with Molecular Diagrams and Heatmaps

Cyto-Safe 1.0 is equipped with explainable AI molecular diagrams and heatmaps to help users better understand the model’s output. The molecular diagram displays the molecule contoured in either red or green, where red represents a strong influence on the model’s prediction of “cytotoxic” and green indicates a strong influence on the prediction of “non-cytotoxic.” As a case study, we evaluated the structures of two established drugs: Doxorubicin, a well-known chemotherapeutic agent with a cytotoxic mechanism of action, and Ibuprofen, a widely used nonsteroidal anti-inflammatory drug. Importantly, neither of these molecules was included in the training sets for the models.

**Figure 2:**
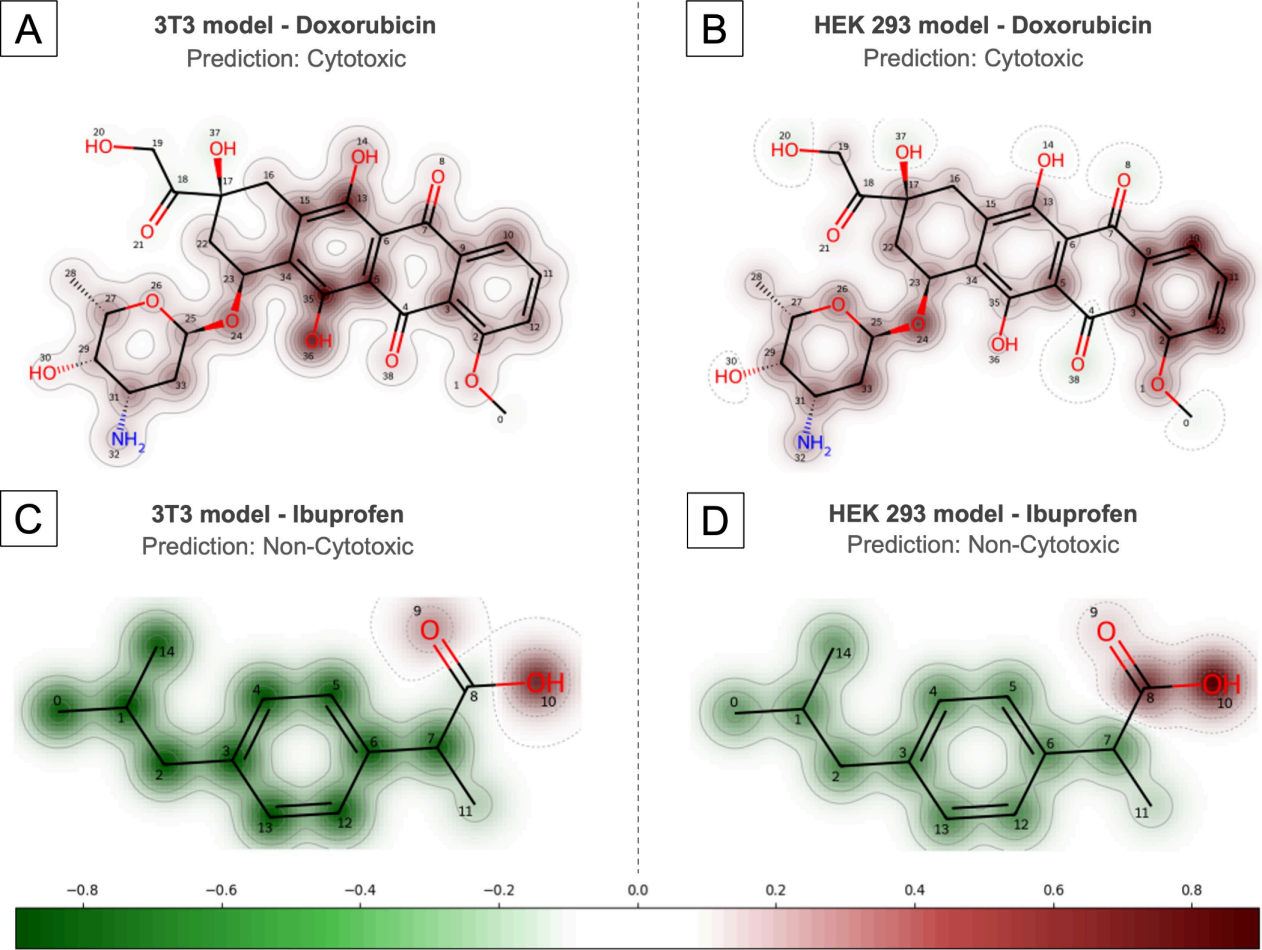
Explainable AI (XAI) molecular diagrams illustrating the model’s predictions for Doxorubicin on the 3T3 (A) and HEK-293 (B) models, and for Ibuprofen on the 3T3 (C) and HEK-293 (D) models. Red contoured regions highlight areas with a strong positive influence on predicted cytotoxicity, whereas green contoured regions indicate a strong positive influence on predicted nontoxicity. The intensity of the contour colors reflects the magnitude of their influence, with darker shades representing a greater impact on the model’s predictions.

Both models classified Doxorubicin as “cytotoxic,” as anticipated, and the molecular diagram reinforced this classification by coloring almost the entire molecule in red in both predictions. In contrast, Ibuprofen was classified as “non-cytotoxic,” with its molecular diagram predominantly highlighted in green, indicating the model’s strong confidence in this prediction (Figure 2). This concordance between the molecular diagrams and the known pharmacological profiles of these compounds underscores the robustness and interpretability of the models.

The atom influence is further illustrated in heatmaps provided in Figures S4–S7 (Supporting Information). These heatmaps employ the same color coding, with red contours indicating atoms that have a strong influence on the model’s prediction of cytotoxicity and green contours representing atoms that have strong influence on the prediction of noncytotoxicity. The intensity of the color contours reflects the strength of the influence, providing users with a detailed understanding of the factors affecting the model’s predictions at both the fragment and atom levels.

The XAI feature of Cyto-Safe is essential for understanding the underlying mechanisms of both models’ predictions. By offering molecular diagrams and atom-wise heatmaps, it identifies specific molecular regions that contribute to either “cytotoxic” or “non-cytotoxic” outcomes. This capability allows users to analyze structural fragments influencing toxicity, facilitating applications in drug design, safety assessments, and compound optimization.

### Limitations

It is important to mention that the Cyto-Safe 1.0, developed to predict cytotoxicity using data from 3T3 and HEK 293 cell lines with the CellTiter-Glo assay, has limitations related to data set characteristics and generalizability. The reliance on only two cell lines - 3T3 (derived from mouse fibroblasts) and HEK 293 (from human embryonic kidney cells) - restricts the model’s applicability to other biological contexts, as these cell lines may not accurately reflect the behaviors of a broader range of cell types. Furthermore, variations in cytotoxicity responses among different cell types - including differences between permanent cell lines and primary cells - underscore the necessity of considering cell type-specific nuances when interpreting cytotoxicity data.^22^

In addition to the cell line limitations, the specificity of the CellTiter-Glo assay complicates the model’s predictions. Different cytotoxicity assays, such as Lactate Dehydrogenase (LDH) release and MTT reduction, may detect varying aspects of cell viability, leading to divergent toxicological profiles. Additionally, variability in experimental conditions - such as cell density, culture medium, and incubation durations - further impacts reproducibility and the model’s reliability across different settings.^23^ The model’s training data are confined to specific incubation times, limiting its applicability for different exposure durations. To enhance the model’s robustness and generalizability, it would be necessary to incorporate a more diverse range of data sets, expanding beyond the current parameters under which it was developed.^24^ Until such advancements are made, users should approach the model’s predictions with a clear understanding of these constraints.

### Comparative Analysis of QSAR Models for Cytotoxicity Prediction

Numerous QSAR models have been developed to predict cytotoxicity, each offering distinct strengths and facing particular challenges. Langdon et al.^25^ employed Bayesian models to achieve cross-assay predictivity; however, the reliance on heterogeneous assay data introduces variability, potentially affecting the consistency and reliability of predictions, and their models lack an interpretability feature.

ProTox 3.0^26^ excels in providing multiend point toxicity predictions by leveraging molecular similarity and machine learning. Its computational efficiency makes it suitable for large-scale screening. However, the model operates as a black box, limiting interpretability and hindering its application in guiding molecular modifications. Yin et al.^27^ addressed the challenge of imbalanced data sets by implementing ensemble learning methods, which deliver strong predictive performance. Despite this, these models are not openly available to the community.

Other notable contributions include the work by Liu et al.,^28^ which focused on predicting microglial cytotoxicity using machine learning models integrated with feature selection and Shapley Additive Explanations. This method provided detailed substructure-level insights, enabling a deeper understanding of toxicological mechanisms. However, its requirement for local installation and computational skills limits its accessibility to users without a strong technical background, and it is limited by only predicting microglial cytotoxicity.

Sun et al.^29^ constructed predictive models based on multiple cell lines using support vector machines (SVMs). While these models achieved high predictive accuracy, they lack an explainable AI feature. Webel et al.^30^ explored the use of deep learning to identify cytotoxic substructures, offering mechanistic insights via Deep Taylor Decomposition. Although their work is promising, it remains exploratory and does not provide a readily available tool for broader use.

Cyto-Safe offers a distinctive approach by integrating prediction accuracy with interpretability and accessibility. Its web-based interface eliminates the need for installations, allowing users from diverse backgrounds to easily conduct predictive analyses. Moreover, Cyto-Safe incorporates Explainable AI (XAI), which generates atom-level heatmaps that elucidate the structural features contributing to toxicity predictions. This transparency not only enhances user confidence but also provides valuable guidance for structural optimization. By supporting multiple data input formats, Cyto-Safe ensures a streamlined experience, catering to both expert and nonexpert users.

In summary, Cyto-Safe bridges the gap between advanced predictive capabilities and practical usability, offering a comprehensive solution for cytotoxicity prediction while addressing the interpretability and accessibility limitations of existing models.

## Conclusions

The Cyto-Safe web application demonstrates substantial efficacy in the binary classification of compounds based on cytotoxicity assessments in 3T3 and HEK 293 cells. Cyto-Safe is distinguished by its user-friendly interface, requiring no programming expertise, and its readiness for immediate deployment. Additionally, it offers transparent explanations of prediction outcomes, representing a significant advancement in the accessibility and usability of cytotoxicity assessment tools. The ongoing development of Cyto-Safe will include the expansion of predictive capabilities to encompass additional cell lines as new, high-quality data becomes available.

However, it is important to recognize the limitations of the model’s predictive capabilities, as they are influenced by the specific experimental conditions used during training. The reliance on 3T3 and HEK 293 cell lines, along with defined incubation times, restricts the model’s generalizability to other cell lines, assays, and exposure durations. To improve its robustness and generalizability, the model should be expanded to include a broader range of data sets, cell lines, assays, and incubation times.

In conclusion, Cyto-Safe is a valuable resource for both the scientific community and industry, facilitating the toxicity evaluation of drug candidates. The tool is freely accessible at http://insightai.labmol.com.br/, enabling users to leverage its capabilities to optimize drug development processes.

## Data Availability

All molecular structures used for each data set modeled are provided in the Supporting Information. The workflows used to calculate descriptors, split the data, train, and validate the models are available at https://github.com/LabMolUFG/cheminformatics_pipeline. The models are available at https://github.com/LabMolUFG/cytosafe.

## Supporting Information Available

Full data sets for the training and test for 3T3 and HEK 293 cell lines (XLSX)
Supplementary methods, results and figures including model’s hyperparameters, clustering and chemical space analysis, applicability domain threshold definition and Explainable AI heatmaps (PDF)
Data sets of compounds clustered by structural similarity (XLSX)
