# Supplementary material for: Cyto-Safe: A Machine Learning Tool for Early Identification of Cytotoxic Compounds in Drug Discovery
Source: J Chem Inf Model. 2024 Dec 11;64(24):9056–62. doi: 10.1021/acs.jcim.4c01811 (PMC11684008; doi:10.1021/acs.jcim.4c01811)
Supplement: Supplementary file 2 — ci4c01811_si_002.pdf [file ci4c01811_si_002.pdf]

Supporting Information

Cyto-Safe: A Machine Learning Tool for Early Identification of Cytotoxic compounds in Drug Discovery

Francisco L. Feitosa[1-3]; Victoria F. Cabral[1-3]; Igor H. Sanches[1-3]; Sabrina Silva-Mendonca[1-3]; Joyce V. B. Borba[1-3]; Rodolpho C. Braga[4]; Carolina Horta Andrade[1-3]*

[1] Laboratory for Molecular Modeling and Drug Design (LabMol), Faculdade de Farmácia, Universidade Federal de Goiás, Goiânia, Goiás, Brazil.
[2] Center for the Research and Advancement in Fragments and molecular Targets (CRAFT), School of Pharmaceutical Sciences at Ribeirão Preto, University of São Paulo, Ribeirão Preto, SP, Brazil.
[3] Center for Excellence in Artificial Intelligence (CEIA), Institute of Informatics, Universidade Federal de Goiás, Goiânia, 74605-170, GO, Brazil.
[4] InsilicAll Inc., São Paulo, SP, Brazil.

Corresponding Author

* Address for correspondence: Laboratory for Molecular Modeling and Design, Faculdade de Fármacia, Universidade Federal de Goiás, Goiânia, GO, 74605-170, Brazil; Telephone: +55 62 3209-6451; E-mail: carolina@ufg.br

Table of contents

Supplementary Methods and Results 3

Hyperparameters selected 3

Activity cliffs analysis 4

Analysis of Chemical Space 5

Supplementary Figures 5

Figure S1 – Chemical space analysis of 3T3 datasets........ 5

Figure S2 – Chemical space analysis of HEK 293 datasets........ 6

Structural Diversity and Applicability Domain Threshold 6

Figure S3 - Distribution of Tanimoto Similarities within the training set for compounds tested on 3T3 (left) and HEK-293 (right) cell lines........ 6

Explainable AI (XAI) heatmaps for the case study 7

Figure S4 – Heatmap contribution of 3T3 model's prediction for Doxorubicin. 7

Figure S5 – Heatmap contribution of HEK 293 model's prediction for Doxorubicin. 7

Figure S6 – Heatmap contribution of 3T3 model's prediction for Ibuprofen. 8

Figure S7 – Heatmap contribution of HEK 293 model's prediction for Ibuprofen. 8

Supplementary References........ 9

Supplementary Methods and Results

Hyperparameters selected

3T3 model:

```
{'boosting_type': 'gbdt',
 'class_weight': None,
 'colsample_bytree': 0.838657087222196,
 'importance_type': 'split',
 'learning_rate': 0.058602817860809286,
 'max_depth': 256,
 'min_child_samples': 20,
 'min_child_weight': 0.001,
 'min_split_gain': 0.0,
 'n_estimators': 3897,
 'n_jobs': -1,
 'num_leaves': 64,
 'objective': None,
 'random_state': 0,
 'reg_alpha': 1e-09,
 'reg_lambda': 0.005217445896529637,
 'subsample': 0.8532738462167552,
 'subsample_for_bin': 200000,
 'subsample_freq': 10,
 'reg_sqrt': True}
```

HEK 293 model:

```
{'boosting_type': 'gbdt',
 'class_weight': None,
 'colsample_bytree': 0.4365853826089555,
 'importance_type': 'split',
 'learning_rate': 0.10987760679510937,
 'max_depth': 99,
 'min_child_samples': 20,
 'min_child_weight': 0.001,
 'min_split_gain': 0.0,
 'n_estimators': 4516,
 'n_jobs': -1,
 'num_leaves': 392,
 'objective': None,
 'random_state': 0,
 'reg_alpha': 0.05622601988408812,
 'reg_lambda': 0.2105202460086872,
 'subsample': 0.7476139566402403,
 'subsample_for_bin': 200000,
 'subsample_freq': 6,
 'reg_sqrt': False}
```

Activity cliffs analysis

We performed a clustering analysis to identify pairs of compounds that have a high degree of similarity but belong to different activity classes (e.g., toxic vs. Non-toxic). The methodology employed for clustering and calculating proportions involved several systematic steps. Initially, molecular fingerprints (ECFP4) were computed for all compounds using their SMILES representations to encode structural features. These fingerprints served as input for the clustering algorithm BitBIRCH[1], which grouped structurally similar compounds based on hierarchical subcluster relationships. The resulting clusters were assigned unique identifiers, and each compound was mapped to its respective cluster. Subsequently, the consistency of biological outcomes (cytotoxic or non-cytotoxic) within each cluster was assessed by analyzing the distribution of outcomes. Clusters were classified as having "homogeneous outcomes" when all compounds shared the same activity and as "heterogeneous outcomes" when compounds exhibited both activities. Finally, the proportions of classified clusters were calculated for each cell type, providing insights into the structural similarity and activity distribution of compounds in the dataset.

BitBIRCH uses a new similarity index known as instant similarity (iSIM)[2] to process binary fingerprints, enabling the application of Tanimoto similarity while reducing memory requirements. Instead of utilizing each compound's features directly, BitBIRCH calculates cluster features (CF) and employs them for clustering, enhancing both efficiency and effectiveness in managing large datasets.

As a result, 5,715 homogeneous and 765 heterogeneous clusters were found for 3T3 cells, and HEK293, 9,201 homogeneous and 1,402 heterogeneous clusters. The heterogeneous clusters (probably representing activity cliffs) represent 11.8% and 13.2% of the training set, respectively. These results indicate that while most clusters show uniform outcomes, a small fraction contains compounds with diverging outcomes, reflecting potential activity cliffs and representing challenging regions for machine learning models to generalize, thus enriching the dataset's complexity and reliability for predictive modeling.

Analysis of Chemical Space

We conducted a chemical space analysis of the datasets to visualize and interpret the distribution of the chemical compounds. We utilized t-distributed stochastic neighbor embedding (t-SNE)[3], available as a module *sklearn.manifold.TSNE* on scikit-learn[4], to transform the original 1024-bit molecular fingerprint matrix into a two-dimensional coordinate system. This transformation facilitated the visualization of complex, high-dimensional data by reducing it to a more interpretable form. We then plotted these coordinates into a scatter plot to examine the spatial distribution and clustering of compounds. To assess the impact of data representation on the analysis, we tested the different under sampling ratios conducted previously. This approach allowed us to evaluate how varying the sample size affected the clustering patterns and overall visualization of the chemical space. Figures S1 and S2 show the chemical space analysis of the 3T3 and HEK 292 datasets, respectively.

Supplementary Figures

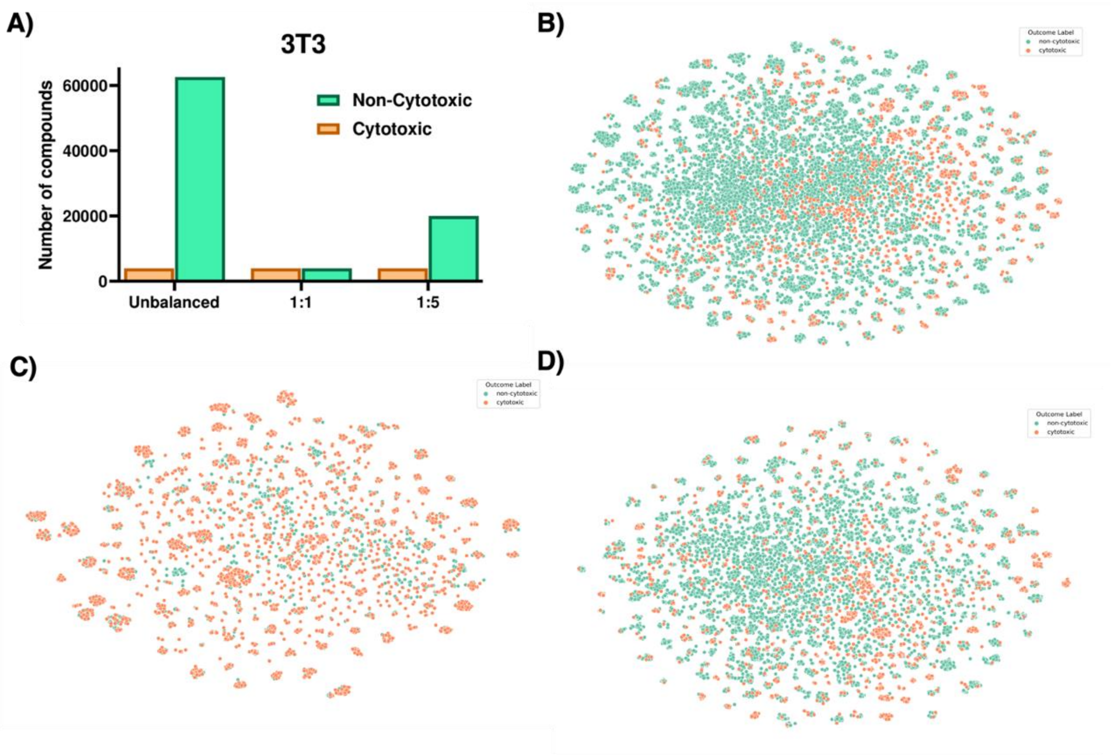

Figure S1 – Chemical space analysis of 3T3 datasets: A) Distribution of compounds; B) Visualization of unbalanced data; C) Visualization of a 1:1 under-sampling proportion; D) Visualization of a 1:5 under-sampling proportion. Note: Green indicates non-cytotoxic compounds, while orange represents cytotoxic compounds.

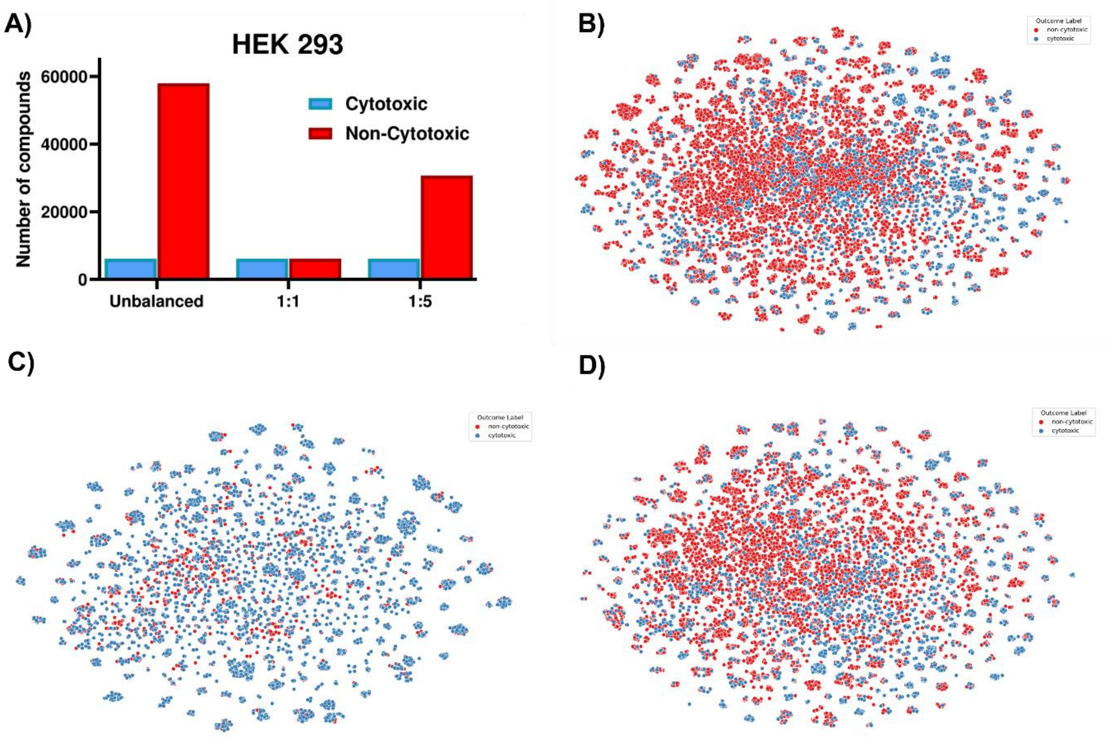

Figure S2 – Chemical space analysis of HEK 293 datasets: A) Distribution of compounds; B) Visualization of unbalanced data; C) Visualization of a 1:1 under-sampling proportion; D) Visualization of a 1:5 under-sampling proportion. Note: Green indicates non-cytotoxic compounds, while orange represents cytotoxic compounds.

Structural Diversity and Applicability Domain Threshold

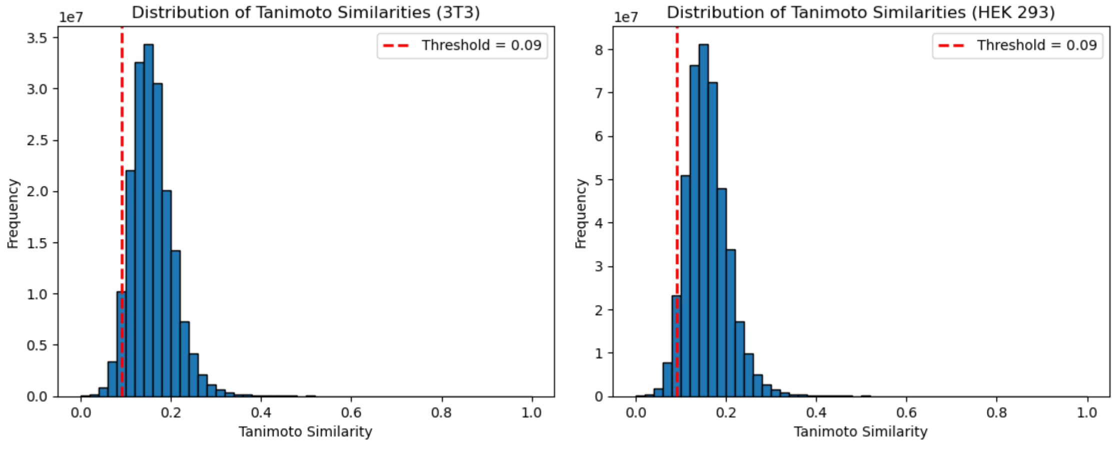

Figure S3 - Distribution of Tanimoto Similarities within the training set for compounds tested on 3T3 (left) and HEK-293 (right) cell lines. The red dashed line represents the threshold (Tanimoto similarity = 0.09) corresponding to the 5th percentile of the similarity distribution.

Explainable AI (XAI) heatmaps for the case study

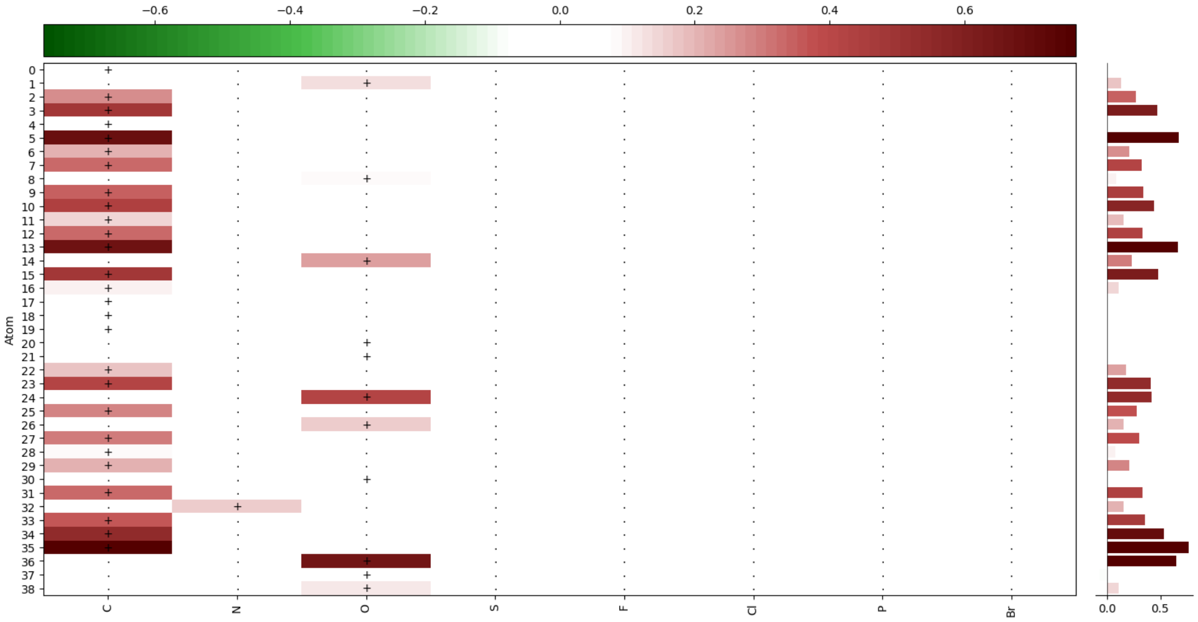

Figure S4 – Heatmap contribution of 3T3 model's prediction for Doxorubicin. Atoms with a positive influence on cytotoxicity are highlighted in shades of red, while atoms with a positive influence on non-cytotoxicity are highlighted in shades of green. The intensity of the color corresponds to the strength of the influence. The *y*-axis corresponds to the atom ID, and the *x*-axis represents the atom type.

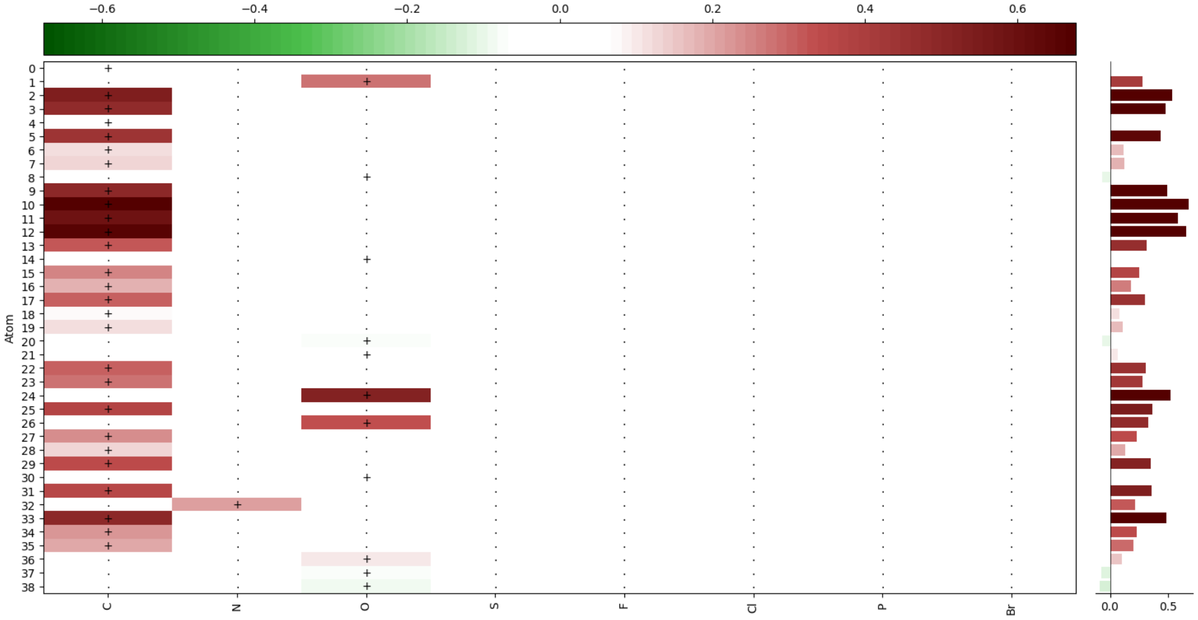

Figure S5 – Heatmap contribution of HEK 293 model's prediction for Doxorubicin. Atoms with a positive influence on cytotoxicity are highlighted in shades of red, while atoms with a positive influence on non-cytotoxicity are highlighted in shades of green. The intensity of the color corresponds to the strength of the influence. The *y*-axis corresponds to the atom ID, and the *x*-axis represents the atom type.

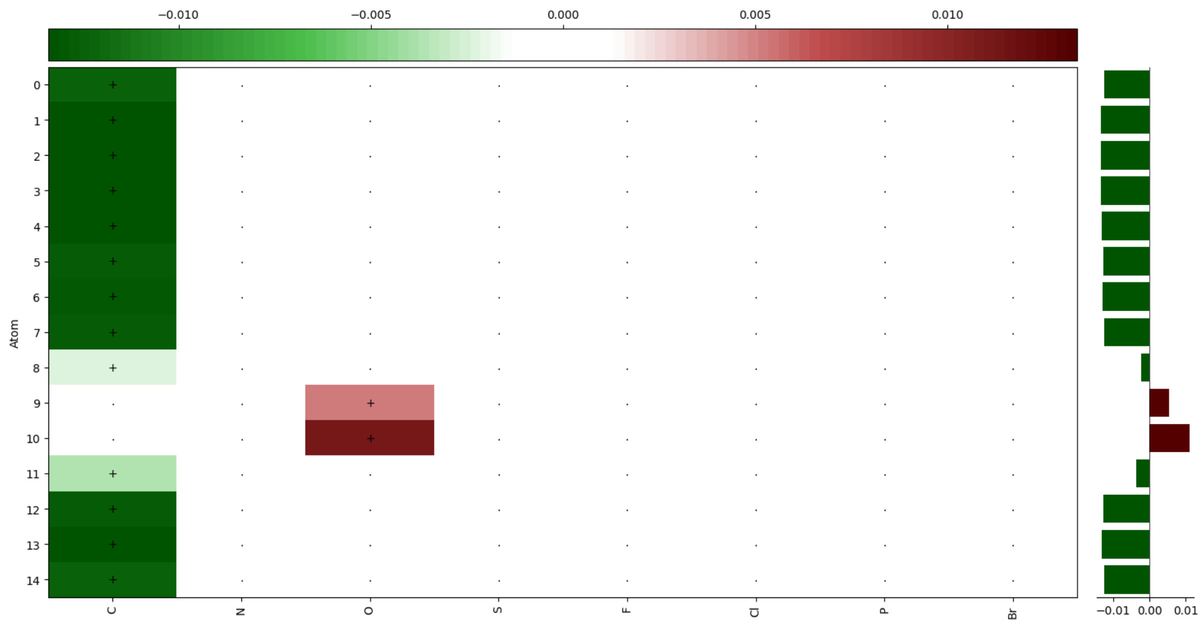

Figure S6 – Heatmap contribution of 3T3 model's prediction for Ibuprofen. Atoms with a positive influence on cytotoxicity are highlighted in shades of red, while atoms with a positive influence on non-cytotoxicity are highlighted in shades of green. The intensity of the color corresponds to the strength of the influence. The *y*-axis corresponds to the atom ID, and the *x*-axis represents the atom type.

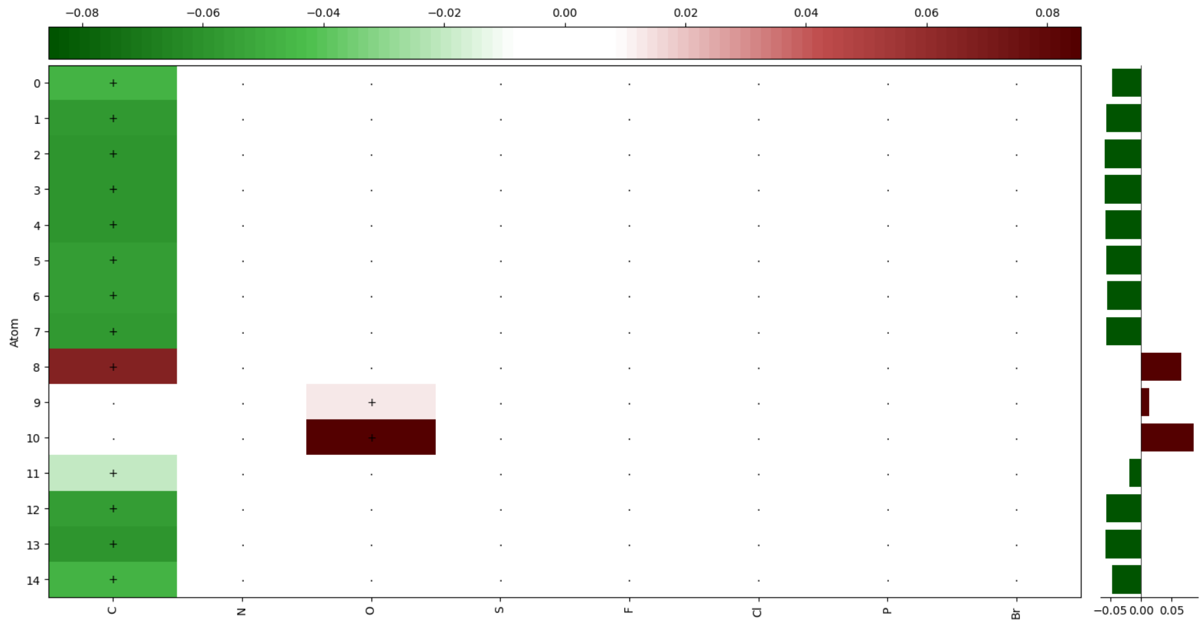

Figure S7 – Heatmap contribution of HEK 293 model's prediction for Ibuprofen. Atoms with a positive influence on cytotoxicity are highlighted in shades of red, while atoms with a positive influence on non-cytotoxicity are highlighted in shades of green. The intensity of the color corresponds to the strength of the influence. The *y*-axis corresponds to the atom ID, and the *x*-axis represents the atom type.

Supplementary References

(1) Pérez, K. L.; Jung, V.; Chen, L.; Huddleston, K.; Miranda-Quintana, R. A. Efficient Clustering of Large Molecular Libraries. August 10, 2024. https://doi.org/10.1101/2024.08.10.607459.

(2) Pérez, K. L.; Kim, T. D.; Miranda-Quintana, R. A. ISIM: Instant Similarity. *Digit Discov* **2024**, *3* (6), 1160–1171. https://doi.org/10.1039/D4DD00041B.

(3) van der Maaten, L.; Hinton, G. Visualizing Data Using T-SNE. *Journal of Machine Learning Research* **2008**, *9* (86), 2579–2605.

(4) Scikit-Learn TSNE Module. https://scikit-learn.org/dev/modules/generated/sklearn.manifold.TSNE.html.
